# Decayed, missing and filled teeth index and dental anomalies in long-term survivors leukaemic children: A prospective controlled study

**DOI:** 10.4317/medoral.17955

**Published:** 2012-08-28

**Authors:** Dorina Lauritano, Massimo Petruzzi

**Affiliations:** 1 Department of Neuroscience University of Milano- Bicocca - Italy; 2 Department of Metodologie Mediche e Tecnologie Chirurgiche - University of Bari - Italy; 3 ….

## Abstract

Objective: The aim of this prospective controlled study is the comparison between long-term children survived leukaemia and a control group in terms of the decayed, missing or filled permanent teeth (DMFT) and dental anomalies. Study design: Fifty-two long term children survived leukaemia, aged from 8 to 15 years (27 females, 25 males; mean age 11.5 years) were evaluated for the possible effects of the anti-leukaemic therapy on dental development and compared to a control group of 52 healthy children (27 females, 25 males, mean age 11 years). All long-term children who survived were at least 24 months in continuous complete remission. The study of the dental status with a routine oral examination and panoramic radiographs was performed. The DMFT (recorded according to the WHO criteria) and dental anomalies were registered and evaluated. Results: The results of this study evidence that long-term children survived leukaemia, in comparison with the control group, have an higher risk to develop dental caries and show a greater severity of dental anomalies including V-shaped roots, dental agenesis, microdontia, enamel dysplasias. Conclusions: Paediatric patients with haemathological diseases require a special attentions in dental care in addition to the antineoplastic treatment. Therefore, oral hygiene and oral health can be maintained thanks to a close cooperation between the paediatric oncohaematologists, pediatrics dental surgeons and dental hygienists.

** Key words:**Paediatric leukaemia, dental anomalies, children.

## Introduction

The rate of children who survived cancer, has considerably improved and a serious effort has been made to avoid the late and side effects of antineoplastic therapies (1). The oral cavity in malignant diseases treated adolescents and young adults shows frequently the signs of the past disease and its anti-neoplastic therapy. The acute leukaemias in children are a classic example of this assertion. Leukaemia constitutes approximately 30% of all childhood cancers and acute lymphoblastic leukaemia (ALL) is the most common type of malignancy (2). On the other hand, acute myeloblastic leukaemia (AML) is a group of malignant bone marrow neoplasms of myeloid precursors of white blood cells. AML is rare and represents approximately 5% of all childhood leukaemias and has an incidence of 1.6 - 2.2 per million per year (3).

The treatment of haematological malignancy involves chemotherapy, radiotherapy and/or bone marrow transplants: this therapeutic approach can cause a real stigmata in the survived patients and can affect their quality of life. The data about oral and dental health of paediatric patients affected by acute leukaemias and subsequently healed, are sparse and their levels of evidence is not always adequate. (4-9) The aim of this prospective controlled study is to describe the oral and dental status of long term childhood leukaemia survivors followed-up at the dental clinic of university of Milan-Bicocca, compared with a control group.

## Material and Methods

Fifty-two children in long-term remission, previously ALL and AML treated at the Division of Haematology-Oncology, Department of Paediatrics of Milano Bicocca University, were included in this prospective study. The survey was performed in the dental clinic of university of Milan-Bicocca. A written consent was given by each patient. The study has been conducted according to e-thical principles of the declaration of Helsinki.

Demographic data, leukaemic treatment regimen, duration of leukaemia remission and type of leukaemia (ALL or AML) were recorded for each patient.

The DMFT (Decayed, Missing, Filled Teeth) score was recorded at least 24 months after the end of leukaemic treatment regimen, according to WHO recommendations. Caries lesions were scored by two blinded calibrated examiners who worked in pair: the first visited and the second collected data. Dental anomalies as microdontia, enamel hypoplasia, dental agenesis and V-shaped roots were assessed according to clinical and radiological examination. A panoramic X-ray was performed in all fifty-two cases.

Children did not receive any fluoride supplement or chlorhexidine treatment during or after the leukaemic treatment.

Moreover, for each patient, age and sex matched control was chosen randomly among school children with a similar so-cio-economic background attending at dental clinic of university of Milan-Bicocca.

-Statistical analysis

Data were analysed using statistical software (STATA version 10.1. USA). Chi-square test was used to compare the prevalence of the DMFT index and dental anomalies in the patients and control group. Differences and correlations were considered signifi-cant if p<0.05.

## Results

Of the 52 patients enrolled 25 were men and 27 women and the mean age was (mean11.5 ±3.5 years). Thirty-nine patients were affected by ALL, the remaining ones were affected by AML. The mean duration of leukaemia remission was 60± 24 months.

Patients were treated according to Italian Association of Paediatric Hematoncology (AIEOP) (10) standard protocol, showed in  Table 1.

**Table 1:**
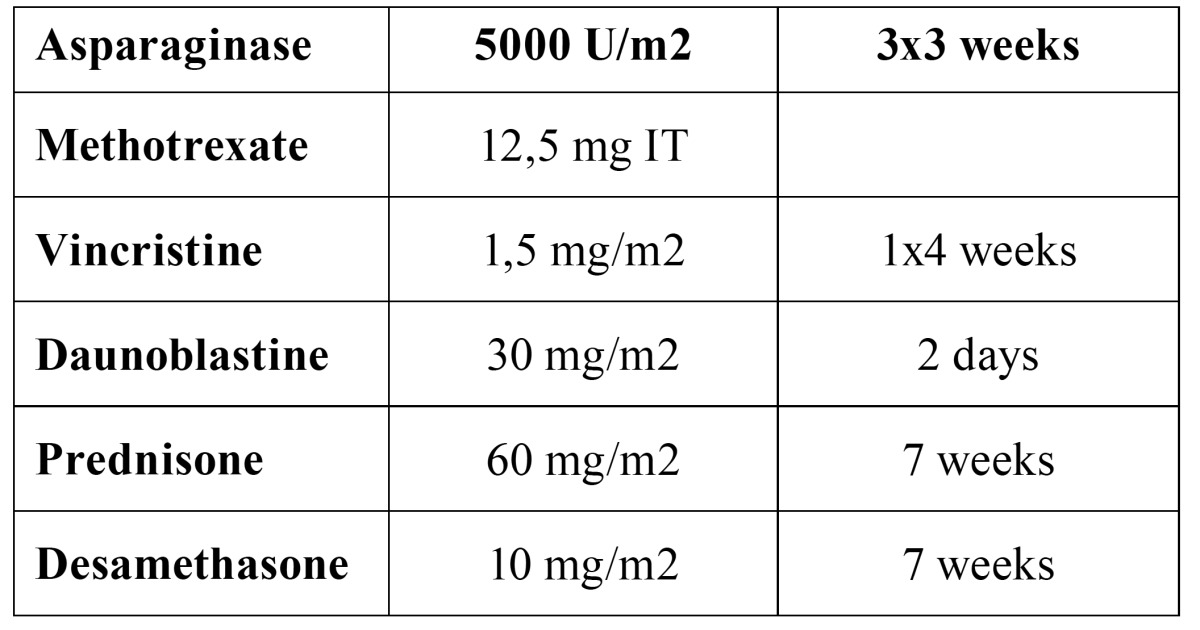
Italian Association of Paediatric Hematoncology(AIEOP) protocol.

Seven patients with ALL received cranial irradiation (18 Gy) in addition to chemotherapy and cytotoxic treatment.

Data about DMFT and dental anomalies in patients and control group are reported in Table 2.

**Table 2:**
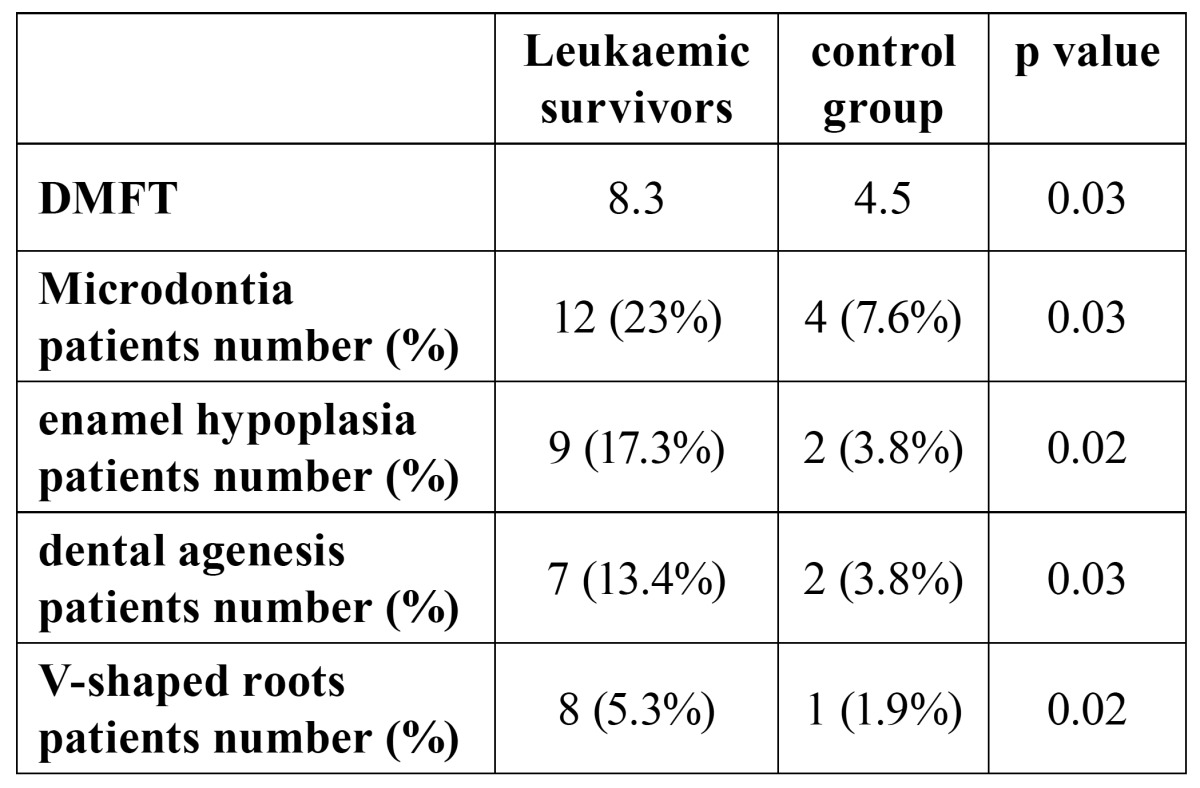
DMFT and Dental anomalies in study and control group.

DMFT was significantly higher in the leukaemic survivors than in the control group (8.3 versus 4.5; p=0.03). Upper incisors, canine and premolars were the most frequent microdontic teeth: twelve leukaemic survivors (23%) showed this dental anomaly while only four subjects (7.6%) in the control group, were positive. The difference was statistically significant (p=0.03). Ena-mel hypoplasia was found in nine leukaemic survivors (17%) mainly on the upper lateral incisors and molars. In the control group we found only two (3.8%) subjects affected by enamel hypoplasia. Also in this case, the difference was significant (p=0.02). Seven leukaemic survivors (13%) showed dental agenesis compared to two subjects (3.8%) in the control group (p=0.03). V-shaped roots (above all lower incisors) were found in eight (15.3%) leukaemic survivors compared with only one subject in the control group. The calculated diffe-rence was statistically significant (p=0.02).

## Discussion

The aim of this study was to investigate the DMFT index and the dental anomalies caused by ALL and AML treatment. To the best of our knowledge, this is the first prospective study on this topic, conducted in Italy in the last 10 years. In paediatric leukaemic survivors, the DMFT index is worse than in the control group. Dental anomalies as hypoplasia, agenesis observed in all ALL and AML treated patients are more frequent than in healthy subjects. The incidence of anomalies in structure, number, size, and shape of teeth are in agreement with the data reported in other studies (4-9). We found additional evidences according to which the dental development is affected by the leukaemia treatment, apart from the patients who were undertaken chemotherapy, radiotherapy, bone marrow transplantation or a combination of these (11-14). Moreover, this study used a matched control group other than siblings in order to reduce the number of variables that might affect results, such as hereditary factors, familiar, diet and hygiene habits.

As far as we know, the DMFT score were rarely investigated in patients with haematological disease. Our results suggest that previous antineoplastic therapies have a negative impact on the dental status of the patients and confirm the observations of other authors who reported an increased DMFT value in leukaemia treated children (15,16).

The negative impact of cytostatic drugs on the oral mucosa as well as the poor oral hygiene, observed during the chemotherapy, are the main causes of the oral health decline. Reduced salivary flow induced changes in the spectrum of bacteria colonizing the oral cavity and favoured caries-related microflora (17). Another consequence of hyposalivation is the excessive use of soft drinks containing sugar, a further risk factor for caries development. Swallowing may become difficult in the cancer period due to the treatment related mucositis, the ulcer caused by Herpes Simplex Virus type 1 infection and the oral bleeding (18). For this reasons, cariogenic soft and sweet food are more frequently chosen. Moreover, mucosal and dental problems are often underestimated by the patients and their parents because of the severity of the underlying haematologic disease. Therefore, regular dental checkups and related treatments remain often neglected. Even some dentist may be reluctant to provide an appropriate treatment for children with cancer because of unjustified fear of serious infection or bleeding. It is necessary the paediatric dentists make regular inspections of the oral conditions of children at least 24 months after healing in order to avoid and prevent dental and mucosal diseases related to leukaemia and its treatment. Other studies documented various pathologic oral and dental malformations in patients treated for malignant diseases: V-shaped roots, microdontia, and agenesis were the most common findings in addition to enamel hypoplasia (4-9,13,14).

Children affected by haemathological diseases requires special attentions and dental care in addition to the antineoplastic treatment. Oral hygiene and oral health can be maintained thanks to a close cooperation between the paediatric oncohaematologists, paediatrics, dental surgeons and dental hygienists. Our experience suggests that the side effects of antineoplastic therapy are unavoidable. However, it is important to increase the quality of life of children affected by ALL and AML in order to prevent the detrimental impact on the oral health.
